# Relationships between pesticides, polychlorinated biphenyls, blood parameters and oxidative stress of white stork *Ciconia ciconia* chicks in Poland

**DOI:** 10.1007/s11356-024-34072-5

**Published:** 2024-06-26

**Authors:** Joachim Siekiera, Łukasz Jankowiak, Artur Siekiera, Monika Ostaszewska, Leszek Jerzak, Mariusz Kasprzak, Mateusz Ciepliński, Piotr Kamiński, Martyna Frątczak, Piotr Tryjanowski

**Affiliations:** 1 Analytical Research and Development Laboratory, Chespa Sp. Z O.O, Pr. Fr. Dusza St. 5, 47-303 Krapkowice, PL Poland; 2 Institute of Biology, University of Szczecin, Wąska 13, 71415 Szczecin, Poland; 3 Department of Nature Protection, Institute of Biological Sciences, University of Zielona Góra, Prof. Z. Szafran St. 1, 65-516 Zielona Góra, PL Poland; 4 Department of Zoology, Faculty of Biological Sciences, University of Zielona Góra, Prof. Z. Szafran St. 1, 65-516 Zielona Góra, PL Poland; 5 Department of Medical Biology and Biochemistry and Department of Ecology and Environmental Protection, Collegium Medicum in Bydgoszcz, Nicolaus Copernicus University in Toruń, M. Skłodowska-Curie St. 9, 85-094 Bydgoszcz, PL Poland; 6 Department of Biotechnology, Faculty of Biological Sciences, Institute of Biological Sciences, University of Zielona Góra, Prof. Z. Szafran St. 1, 65-516 Zielona Góra, PL Poland; 7 Department of Zoology, Poznań University of Life Sciences, Wojska Polskiego 71C, 60-625 Poznań, Poland

**Keywords:** Storks, Birds, Blood morphology, Blood biochemistry, Environmental pollution, Ecotoxicology, Pesticides

## Abstract

The white stork *Ciconia ciconia* is a bird species located at the top of the trophic pyramid in grassland and wetland ecosystems. This charismatic species is susceptible to pesticides and their environmental residues. In 2016, we collected blood samples from 114 white stork chicks across Western and Southern Poland. Chicks were sexed by molecular analysis and aged by development pattern. We studied the relationship between the concentration of pesticides (beta-HCH, heptachlor, aldrin, endrin, 4.4′-DDD, 4.4′-DDE and 4.4′-DDT) and of PCB in the chicks’ blood with blood morphology and biochemistry parameters in the blood. The *mean* (± *SD*) values of concentrations of above detection level pesticides were: for (1) beta-HCH 4.139 ± 19.205; (2) 4.4′-DDE 9.254 ± 91.491 and additionally (3) PCB 16.135 ± 44.777 ppb. We found negative relationships between beta-HCH and oxidative stress enzyme activity in the blood, between beta-HCH and leukocyte concentration and between 4.4′-DDE and catalase activity. We also found a positive relationship between the concentration of pesticides in blood and the age of chicks. Interestingly, we found a higher concentration of PCB in the blood of male stork chicks than in female stork chicks. We provide more evidence that the presence of pesticides in the environment can be a strong stress factor, shaping the health status of birds.

**Supplementary Information:**

The online version contains supplementary material available at 10.1007/s11356-024-34072-5.

## Introduction

White storks, like many bird species using agricultural landscapes as a breeding habitat, can be chronically exposed to pesticides and other agrochemicals (Newton 2004; Mitra et al. 2021). Within the European Union about 400 pesticides are still allowed to be used (European Commission 2023). Although they are perceived to be relatively safe, standard tests of their toxicity do not consider their long-term exposure effects, build-up of pesticide residues in the food chain and combined effects of several pesticides (Storck et al. 2017). Due to their high position in the food chain, white storks are chronically exposed to different types of pesticides, mainly by ingestion of contaminated prey (amphibians, earthworms, snails, fish, rodents, etc.) and plant seeds. Exposure of birds to agrochemicals can also occur through inhalation of contaminated air or direct contact with contaminated soil and water (Pinowski et al. 1991). It has been shown that pesticides can negatively affect birds and cause disruption of functions of their reproductive, endocrine and nervous systems (Shimshoni et al. 2012; Vos et al. 2000). In recent years, serious declines in populations of white storks and many other waterfowl and farmland bird species have been noted (Newton 2004; Tryjanowski et al. 2005; Burns et al. 2021; Wuczyński et al. 2021). A higher mortality rate among young birds has been linked to the contamination of the environment (Tryjanowski et al. 2005).

However, there is still a need for studies contributing to a better understanding of the influence of agrochemicals on bird health and survival. This can be achieved by finding a correlation between the presence of pesticides in the environment with those in bird tissues and with blood morphology or biochemical parameters. Blood morphology and biochemical parameters are determined by many internal and external environmental factors, such as age, sex, ambient temperature, reproductive status, parasite load, food reserves and contaminants (Puerta et al. 1995; Benito et al. 1999; Jerzak et al. 2010). Examining the blood parameters of birds can be a source of important information regarding their condition and health status. Particularly in the context of our study, changes in haematological and biochemical blood parameters may indicate the contamination of birds with pollutants (Benito et al. 1999; de la Casa-Resino et al. 2015; Carravieri et al. 2017). This knowledge can contribute to the more effective monitoring of environmental pollution and the protection of farmland birds (Blázquez et al. 2006; Orłowski et al. 2006).

There is still not enough information about the connection between blood parameters, the physical condition of birds and environmental pollution with pesticides. However, some interesting investigations on this topic have been undertaken. Blázquez et al. (2006), de la Casa-Resino et al. (2015) and Pérez-López et al. (2016) studied the levels of different inorganic elements (Pb, Hg and As) and persistent chlorinated pollutants (including polychlorinated biphenyls (PCBs) and organochlorine pesticides (OCPs)) in blood and plasma of white stork chicks from NW Spain. These studies determined that the levels of the studied elements and pollutants were generally lower than the risk thresholds for birds but were a useful baseline for biomonitoring the levels of measured contaminants in the study areas. These baseline references can also be found for physiological blood parameters of the white stork (Szabó et al. 2010) and the Oriental white stork *Ciconia boyciana* (Han et al. 2016). Other, notable, studies presented data on the wider topic of the influence of pollutants on white stork physiology (Baos et al. 2006; Blas and Baos 2008; Muñoz-Arnanz et al. 2008; Sáez et al. 2008a).

The aims of the current paper were to investigate the concentrations of selected, widely used, pesticides: beta-HCH, heptachlor, aldrin, endrin, 4.4′-DDD, 4.4′-DDE, 4.4′-DDT and PCB in the blood of white stork chicks from Western and Southern Poland, and to identify any relationships between these contaminants and blood morphology, biochemistry, age and sex of the chicks.

Due to a much higher number of studied parameters than in similar studies (Muñoz-Arnanz et al. 2008; de la Casa-Resino et al. 2015), we used multivariate statistics. We showed not only simple interactions between pesticide concentration in blood, blood morphology and biochemistry but also the relation between pesticide concentration in blood and oxidative stress enzymes in white stork chicks (Kamiński et al. 2009a, b).

## Material and methods

### Study area

Data were collected in the Odra River valley in SW Poland, at the following sites: Krapkowice industrial area (50°28′N, 17°58′E); Głogów copper manufacturing area (51°39′N, 16°04′E), villages located about 20 km from Zielona Góra (100,000 inhabitants; 51°56′N, 15°30′E), and Kłopot meadows (52°07′N, 14°42′E). The local population of the white stork has declined in recent years (Wuczyński et al. 2021). Based on the location of industry, sites were divided into two simple categories: (1) polluted — Krapkowice and Głogów and (2) unpolluted — Zielona Góra and Kłopot and were used in this way in statistical analyses and throughout this paper.

### Blood sampling procedures

In total, we studied 114 white stork chicks. The age of birds varied from 14 to 54 days after hatching. Age was established according to Kania (1988) and previously tested in the studied populations (Jerzak et al. 2010). Our behavioural observations and physical examination of the chicks suggested that all of them were in generally good physical health. To eliminate potential changes in studied parameters due to a diurnal rhythm, all examinations took place between 1000 and 1200.

Samples for biochemical and morphological analyses were taken from the wing venous blood of birds. Chicks were retrieved from the nest and placed into individual, ventilated cotton sacks. Blood samples (5 ml) were collected via venepuncture of the brachial vein, using a 5-ml syringe rinsed with ethylenediaminetetraacetic acid (EDTA). While obtaining blood samples from the birds the stress factor was reduced by covering the head of the chicks with the ventilated cotton sacks. Blood smears were prepared immediately after collection, using the push-slide technique. Blood smears were then air-dried, fixed in methanol, and stained with the May-Grünwald stain method (Robertson and Maxwell 1990). The rest of the blood samples were kept in a cooler before transporting to the laboratory. After centrifugation, plasma samples were frozen at –20 °C and stored until analysis.

For sexing chicks, the blood was collected in ethylene-diamine-tetra-acetic acid (EDTA) treated plastic tubes and stored at 4 °C. Total genomic DNA was extracted from the whole blood using MasterPure™ DNA Purification Kit for Blood (Epicentre Technologies) following manufacturer’s protocol with a little modification introduced by the current authors. To obtain good quality and high quantity DNA, we used 180 μl of whole blood instead of 360 μl, as recommended by the manufacturer. Volumes of other buffers were the same as in the manufacturer’s protocol. To check the quality of extracted DNA, electrophoresis of total genomic DNA samples was conducted in 1% agarose gels in onefold TBE buffer (10 × TBE: 0.89 M Tris, 0.89 M boric acid, 0.02 M EDTA, pH 8.0) on 120 V for 60 min and stained with ethidium bromine. The concentration of dsDNA in samples was measured using spectrophotometer Nano-Drop (Thermo Scientific, USA). To detect the genetic sex of young white storks, the multiplex PCR method was used. Two sets of primers were used: (1) pair of primers specific for W-chromosome as described by Itoh et al. (1997) for amplification of female specific sequence on W-chromosome in the Oriental white stork; (2) second pair of primers was used in order to replicate the 18S ribosome gene, which serves as a positive internal control of PCR reaction (Itoh et al. 1997). The amplification was performed in 20 μl reaction volume containing approximately 50 ng of genomic DNA, 8 pmol of each primer, 200 μM of each dNTP, 2.0 mM MgCl2 and 1.75 U Taq polymerase (Thermo Scientific Fermentas) in onefold reaction buffer with KCl. The temperature profile of the reaction was denaturation at 94 °C for 2 min, followed by 35 amplification cycles of 80 s at 94 °C, 90 s at 58 °C, 60 s at 72 °C and final extension of 5 min at 72 °C. Amplicons were separated in 2.5% agarose gel in 1 × TBE buffer (10 × TBE: 0.89 M Tris, 0.89 M boric acid, 0.02 M EDTA, pH 8.0) for 120 V during 60 min and stained with ethidium bromine. After electrophoresis, the genetic sex of each white stork chick was determined on the basis of the number of bands in the line of each individual upon agarose gel (see details in Jerzak et al. 2010).

### Laboratory analyses

Collected blood samples were analysed for the presence of pesticide residues. Quality control was used as suggested by the QA/QC Directive (2009/90/EC), with the use of blank samples, duplicate samples and the use of certified reference materials (CRMs). Blank samples were included in each batch of analysis to monitor for any potential contamination during the analytical process. These blanks were consistently found to be free of target analytes, ensuring no cross-contamination occurred. Duplicate analyses of selected samples were conducted to evaluate the precision of the method and demonstrated reproducibility of the results. To further validate our analytical method, CRMs for the analysis of beta-HCH, 4.4′-DDE and Aroclor 1242 obtained from Sigma-Aldrich/Merck KGaA were used. The purity of analytical standards was > 99%. Aroclor 1242 was used as the reference for PCB analysis, with LOD at 1.581 ppb. These CRMs were analysed in parallel with the samples and provided a benchmark for comparison and ensured the accuracy of our analysis.

An Eppendorf centrifuge 5430 was used to extract the samples and precipitate the proteins. Vortex ZX4 Velp Scientifica was used for mixing. Serum samples (0.5 ml) were quenched with 0.5 ml of methanol to precipitate proteins. Centrifugation was conducted for 5 min at 17,500 rpm. After centrifugation, the supernatant was removed and poured into a centrifuge tube (15 ml volume) containing 2 ml of n-hexane. The tubes were shaken on a vortex shaker for 3 min at 3000 rpm. The hexane phase was transferred to a 1.5 ml vial.

The quantitative analysis of pesticides was carried out using a Shimadzu GC-2010 Pro gas chromatograph equipped with an MS/MS detector. The chromatograph was equipped with a ZB-Semi Volatiles capillary column (internal diameter 0.18 mm × 20 mm, layer thickness 0.18 µm, Phenomenex, USA). The oven temperature was held at 70 °C for 0.75 min and then increased by 5 °C/min to 250 °C and later by 20 °C/min to 300 °C and held for 2 min. The injection port and interface were kept at 260 °C. Helium was used as the carrier gas. AOC-20i (Shimadzu) was used for injection. The spray volume was 1 µl. The quantification of pesticides was performed using the multiple reaction monitoring (MRM) method. Standard curves were prepared by the gravimetric method using an analytical balance from Sartorius. N-hexane was used for dilutions.

The morphology and biochemistry of the chicks’ blood were analysed. Photos of the blood cells were taken using a Nikon Eclipse Ni microscope with digital camera (Nikon DS-Fi2) and processed using the image software NIS-Elements Basic Research. Biochemical analysis (concentration of total protein, urea, uric acid, triglycerides, total cholesterol, high density lipoprotein (HDL), low density lipoprotein (LDL) and activity of aspartate aminotransferase (ASPAT), and alanine aminotransferase (ALAT)) was performed with the use of ARCHITECT c4000 clinical chemistry analyser (Abbott Diagnostics).

Red blood cell (RBC) and white blood cell (WBC) counts were made using a Bürcer chamber after staining with Natt and Herrick solution in an RBC-diluting pipette (Natt and Herrick 1952). Haematocrit (HCT) was measured using the microhaematocrit method. The haemoglobin (Hb) level was determined with the use of Drabkin’s (1945) colorimetric method. Haematological parameters were assessed using routine manual methods. Total WBC was counted using the Natt and Herrick method (Campbell 2015). Blood smears were analysed under a Nikon Eclipse Ni microscope with a digital camera. Cells were observed under a 1000 × magnification and classified as heterophils, eosinophils, basophils, lymphocytes and monocytes, according to criteria specified by other authors (Lucas and Jamroz 1961; Clark et al. 2009). The heterophil and lymphocyte ratio (H/L ratio) was then calculated by dividing the number of heterophils by the number of lymphocytes (Lentfer et al. 2015).

Examination of superoxide dismutase (SOD) activity in serum was conducted with a commercially available set manufactured by Cayman Chemicals Co. Ltd. (Superoxide Dismutase Assay Kit, Item No. 706002). Examination of catalase (CAT) activity in serum was conducted with the commercially available set manufactured by Cayman Chemicals Co. Ltd. (Catalase Assay Kit, Item No. 707002). Examination of glutathione peroxidase (GPx) activity in serum was conducted with a commercially available set manufactured by Cayman Chemicals Co. Ltd. (Glutathione Peroxidase Assay Kit, Item No. 703102). Examination of GR activity in serum was conducted with a commercially available set manufactured by Cayman Chemicals Co. Ltd. (Glutathione Reductase Assay Kit, Item No. 703202). Ceruloplasmin (CP) concentration was determined using Ceruloplasmin ELISA Kit (Wuhan EIAab Science, Cat. No. E0909h). Examination of malondialdehyde (MDA, lipid peroxidation marker) level in serum was conducted with a commercially available set manufactured by Cayman Chemicals Co. Ltd. (TBARS Assay Kit, Item No. 10009055). All the procedures with the use of commercial sets were performed strictly following the manufacturer’s protocol.

### Statistical analyses

Although samples were analysed for the entire panel of pesticides, only beta-HCH (0.493 ppb), 4.4′-DDE (0.097 ppb), and PCB (1.581 ppb) were found to be above the respective method detection limits and therefore we report results only for these three analytes.

First, we tested data for spatial autocorrelation with Moran’s local indicator (Legendre and Legendre 1998). As a data set, we used geographic position and nest averages of three dependent variables beta-HCH, 4.4′DDE, and PCB. We found a significant spatial correlation for beta-HCH and DDE (Figs. S1 and S2 in Supplementary Materials) but not for PCB (Fig. S3). To deal with spatial autocorrelation, we performed an autoregressive model for HCH and DDE, and predictions from this model were used as a spatial covariate in further analysis (spatial_HCH, spatial_DDE). The spatial autocorrelations and autoregressive models were computed using SAM software (Rangel et al. 2010).

To simplify the environmental variables, we used principal component analysis (PCA). We performed the first PCA on enzyme variables (SOD, CAT, GSH, GPx, GR, MDA and CP). We used the two first axes (PC1 and PC2), which explained a total of 47% of the variance. The correlation chart[matrix?] is shown in the Supplementary Material (Fig. S4 and Table S1). PC1 (hereafter PC1_SOD) was positively correlated with SOD, MDA, GPx, GR and GSH. PC2 (hereafter PC2_CAT) was positively correlated with CAT and CP enzyme.

The second PCA was performed on blood morphology: (RBC, WBC, HCT, Hb, and mean corpuscular volume (MCV), mean corpuscular haemoglobin (MCH), mean corpuscular haemoglobin concentration (MCHC)) and biochemical parameters (total protein, urea, uric acid, triglycerides, total cholesterol, HDL, LDL, ASPAT and ALAT) (Fig. S5 and Table S2 in Supplementary Materials). We chose the first four axes which explained a total of 61% of the variance. PC1 (PC1_biochemical) was positively correlated with ASPAT, ALAT, total cholesterol, TG (triglycerides), protein, urea and uric acid and could be interpreted as a biochemical indicator. PC2 (PC2_morphology) was positively correlated with HCT, Hb and RBC and negatively with monocytes, MCV and MCH, and this variable could be interpreted as a blood morphology indicator. PC3 (PC3_leukocytes) was positively correlated with monocytes, lymphocytes, WBC, eosinophils and basophils and negatively with proteins, and could be interpreted as a leukocyte indicator. PC4 (PC4_nitrogen) was positively correlated with urea, uric acid, TG, basophils and HCT and negatively with WBC, lymphocytes, HDL, LDL and total cholesterol and could be interpreted as a non-protein nitrogen compound indicator.

The concentrations of beta-HCH and 4.4′-DDE were modelled by linear mixed models with censored observations (Kuhn 2021). A linear mixed model was used for PCB (Bates et al. 2014). For each of the models nest ID was a random effect to avoid pseudoreplication because records were obtained from chicks in the same nest. We used PC1_SOD, PC1_CAT, PC1_biochemical, PC2_morphology, PC3_leukocytes, PC4_nitrogen, age of chicks (in days), pollution (two levels: polluted, non-polluted) and sex as independent variables. We started with global models incorporating all variables. The final model was selected using backward stepwise removal of non-significant effects (*P* < 0.05) (Zuur et al. 2009). However, during the selection, age was always present in the model terms because we treated it as a control variable since the concentration of the pesticides could be the result of accumulation during the life of the chicks.

We used R software (R Development Core Team 2018) for analysis. For the mixed modelling approach, we used the “lme4” package (Bates et al. 2014) and for the mixed modelling with censored data, we used the “lme4cens” package. The mean values for censored data were computed using regression on order statistics implemented in “NADA” packages (Lee 2020).

## Results

Overall summaries for pesticide concentration are presented in Table 1.

**Table 1:** Concentrations of studied substances in analysed blood samples of white stork chicks (*n* = 114). Data are presented in ppb (on a wet weight basis)

Concentration	Mean	*SD*	Median
beta-HCH	4.139	19.205	1.783
4.4′-DDE	9.254	91.491	0.129
PCB	16.135	44.777	8.490

For beta-HCH, we found a significant difference between the polluted and non-polluted areas (Table 2). The mean beta-HCH concentration for the polluted areas (6.2502 ± 23.943 *SD*, median = 2.1695) was significantly higher than for the non-polluted areas (1.1751 ± 1.1991 *SD*, median = 1.036). There was a negative relationship of beta-HCH with PC1_SOD, and positive relationships with PC2_CAT, PC3_leukocytes and chick age (Table 2).

**Table 2:** Results of three different models explaining beta-HCH, DDE, and PCB concentration in the blood of white stork chicks^1^

	Estimate	Std. error	*z*	Pr( >|*z*|)
beta_HCH				
(Intercept)	− 1.067	0.304	− 3.51	< 0.001
Pollution (polluted)^2^	0.817	0.048	17.09	< 0.001
PC1_SOD	− 0.042	0.014	− 3.13	0.002
PC2_CAT	0.045	0.018	2.54	0.011
PC3_leukocytes	0.027	0.010	2.68	0.007
Age	0.728	0.194	3.76	< 0.001
spatial_HCH	− 0.169	0.020	− 8.41	< 0.001
DDE				
(Intercept)	− 3.202	0.856	− 3.740	< 0.001
PC2_CAT	− 0.093	0.042	− 2.220	0.026
Age	2.133	0.562	3.790	< 0.001
spatial_DDE	0.110	0.050	2.200	0.028
PCB				
(Intercept)	0.719	0.449	78.721	0.113
Sex (males)^3^	0.103	0.045	98.068	0.024
PC3_leukocytes	− 0.035	0.016	113.776	0.037
Age	0.131	0.300	77.257	0.663

^1^Please note that for beta-HCH and DDE we used a linear mixed model with censored observations. However, for PCB, because we did not have censored observations, a linear mixed model was performed

^2^Binary covariate, with “non-polluted” serving as the reference category

^3^Binary covariate, with “females” serving as the reference category

4.4′-DDE exhibited a negative relationship with PC2_CAT and a positive relationship with chick age (Table 2).

For PCB, we found a significant difference between male and female chicks (Table 2). The mean PCB concentration for male chicks (23.685 ± 64.333 *SD*, median = 8.528) was significantly higher than in female chicks (9.340 ± 4.816 *SD*, median = 8.44). A negative relationship with PC3_leukocytes was evident (Table 2).

## Discussion

Birds encounter pesticides and their residues in many ways. Direct exposure to pesticides may happen when birds enter fields or nearby areas shortly after spraying with agrochemicals (Mineau 2002). Slower but also highly harmful exposure happens through ingesting prey that had contact with pesticides. Pesticides can bioaccumulate, which means that their concentrations in organisms can increase with the levels of the food chain. In effect, birds can ingest large amounts of toxins through their prey (Helander et al. 2008). There is much evidence that birds are harmed by currently used pesticides (Mitra et al. 2021). Declining bird populations have been found to be associated spatially and temporally with pesticide use (Mineau 2002). Moreover, the indirect effects of pesticides negatively influence bird populations. For example, these pesticides, which generally are not directly toxic to exposed birds, can affect invertebrates and other prey, shrinking important food resources of birds (Bright et al. 2008).

Because of the common presence of pesticides and their residues in the environment, monitoring their influence on bird condition and survival is essential (Blázquez et al. 2006; Orłowski et al. 2006; Helander et al. 2008). The white stork population can be particularly sensitive to environmental threats. Population growth in white stork is mainly favoured by high adult survival rates, which compensates for the low-fledging success. However, chick survival is also important for the white stork population (Schaub et al. 2004). Pesticide presence in the environment can affect adult white storks and chicks in different ways. Young birds can be more sensitive to the acute effects of pesticide exposure than adults which may be constantly in contact with some level of toxins and with a possibly higher tolerance to them (Grue and Shipley 1984). Adult birds, on the other hand, can be more prone to the chronic effects of pesticides, caused by a build-up of toxic residues in their tissues (Yohannes et al. 2017; Chen et al. 2008; Damalas and Eleftherohorinos 2011). A better understanding of pesticide risks for chicks and adult white storks is needed. Of special interest are sexual differences in PCB concentrations among chicks, that is perhaps linked to activity of sexual hormones, mainly steroids (Hao et al. 2021). In the case of the stork, changes related to sex may be further modified by sexual differences in haematological blood parameters and the rate of development of males and females (Jerzak et al. 2010; Tryjanowski et al. 2011).

We also highlight the predominant impact of pesticides on stork physiology, affecting enzymatic activity and biochemical processes. Stork chicks exposed to high pesticide levels showed increased lipid peroxidation and intense oxidative modification of proteins (carbonylation) in their blood. Lipid peroxidation and oxidative modification of proteins are initiated by free radicals (ROS), whose availability is largely dependent on the activity of antioxidant enzymes (such as superoxide dismutase, catalase, glutathione reductase, glutathione peroxidase and ceruloplasmin) (Bhattacharya 2015). Antioxidant activity appears to have been depleted in the storks as beta-HCH increased, as is indicated by the negative relationship with PC1 (SOD, GPX and GR) which was estimated by thiobarbituric acid reactants. Pesticides can act as modulators by lowering antioxidant enzymatic activity (Ercal et al. 2001). By this mechanism of action, pesticides can have serious implications for bird health. For example, a high level of lipid peroxidation may be associated with the impairment of membrane-dependent functions of mitochondria and lysosomes in the liver (Barja 1998), while an increased level of protein carbonylation could be linked to pathological changes and premature aging process within organs (Beal 2002). This indicates a dynamic response to oxidative stress, where antioxidant levels fluctuate based on the need to protect against damage to DNA, lipids and proteins. Importantly, our results suggest that, even in an environment without heavy industry, birds faced oxidative stress, potentially induced by factors like transportation and proximity to motorways.

A powerful and relatively easy-to-use tool for monitoring exposure and the influence of pesticides on birds could be the analysis of their blood parameters (Katagi and Fujisawa 2021). As shown in our study, pesticides commonly used in agriculture can be present in the blood of white stork chicks and are linked with their morphological (leukocytes) and biochemical parameters (antioxidants). The results for beta-HCH differed between birds from polluted and non-polluted environments. Similar differentiation has been demonstrated in white storks from various environments in Spain (Blázquez et al. 2006; Blas and Baos 2008; Muñoz-Arnanz et al. 2008; Sáez et al. 2008b; de la Casa-Resino et al. 2015; Pérez-López et al. 2016).

It appears that the pesticide concentrations detected in storks in our study have a similar physiological impact (mainly on the blood parameters of chicks) as in similar experiments conducted in Extremadura (Spain) by Pérez-López et al. (2016). Their study showed that the level of pesticides in the blood of white stork chicks may depend on the concentration of pesticides used in the environment, on the feeding strategy of the parents, as well as on mutual, parallel interactions between individual pesticides. Although in that study, conducted in Spain, no effects were observed in white stork chicks from an area with relatively low pesticide pollution, it was stated that examination of white stork chicks can be useful to assess the biological effects of the high level of pollution in the environment on birds. A similar conclusion can be made from our study. Pesticides (beta-HCH) and leukocytes showed a significant spatial relationship, which was probably related to local pollution level and local population foraging strategies of white storks (Wuczyński et al. 2021). Logically, in all models explaining pesticide concentration, chick age also played an important and statistically significant role, which can be due to the tendency of pesticide concentration in tissues of animals over time of exposure (Baos et al. 2006; Goutner et al. 2011; de la Casa-Resino et al. 2015).

However, not all studies indicate a strong relationship between pesticide pollution of the environment and physiological parameters of birds. For example, in studies from China (Chen et al. 2008; Wang et al. 2016) and the Korean Peninsula (Park et al. 2017), pesticide concentration in tissues of Oriental white storks were relatively low, which indicated that these birds were not unduly influenced by pesticide pollution.

In our study the tested pesticides had a much stronger effect on the values of biochemical parameters in white stork chicks. Pesticide presence in blood was linked to the most important biochemical parameters, especially urea, uric acid, triglycerides, LDL and ASPAT. This mainly involved the pesticides beta-HCH, aldrin, endrin, 4.4′-DDE and 4.4′-DDT (Table S2, Figs. S1–5). The strongest correlations between pesticides in blood and their biochemical parameters were found for white storks from the polluted area.

Moreover, blood leukocyte levels and enzyme levels were correlated with the concentration of pesticides in the blood of white stork chicks. For white stork chicks from polluted areas, there was a strong relationship with the level of pesticides in the blood. Therefore, we can conclude that pesticide pollution in these can influence blood parameters of white stork chicks. This indicates a possible antagonistic effect of pesticide pollution on white stork chick health and nutritional status, which can translate to a lower survival rate. Ultimately, contact with pesticides from a young age (see Jerzak et al. 2010) can strengthen the adaptability of white storks to environmental contaminants. More studies are needed to explain these issues.

### Supplementary Information

Below is the link to the electronic supplementary material.

Supplementary file1 (DOCX 44 KB)

Supplementary file2 (XLSX 91 KB)

Supplementary file3 (DOCX 116 KB)

## Data Availability

Yes. On the e-mail request from Dr. Łukasz Jankowiak, e-mail: jankowiakl@gmail.com.
