# Supplementary material for: Relationships between pesticides, polychlorinated biphenyls, blood parameters and oxidative stress of white stork Ciconia ciconia chicks in Poland
Source: Environ Sci Pollut Res Int. 2024 Jun 26;31(31):43996–4004. doi: 10.1007/s11356-024-34072-5 (PMC11252220; doi:10.1007/s11356-024-34072-5)
Supplement: Supplementary file 1 — Supplementary file1 (DOCX 44 KB) [file 11356_2024_34072_MOESM1_ESM.docx]

**Supplementary materials**

**Tables**

**Table S1.** Pearson correlations between enzymes, and each of enzyme with Principal Component two axes.

| row | column | cor | p |
| --- | --- | --- | --- |
| SOD | CAT | 0.065 | 0.468 |
| SOD | GSH | 0.079 | 0.382 |
| CAT | GSH | 0.002 | 0.980 |
| SOD | GPx | 0.350 | <0.001 |
| CAT | GPx | 0.059 | 0.512 |
| GSH | GPx | 0.235 | 0.008 |
| SOD | GR | 0.299 | 0.001 |
| CAT | GR | 0.037 | 0.678 |
| GSH | GR | 0.182 | 0.042 |
| GPx | GR | 0.292 | 0.001 |
| SOD | MDA | 0.367 | <0.001 |
| CAT | MDA | -0.014 | 0.880 |
| GSH | MDA | 0.280 | 0.001 |
| GPx | MDA | 0.256 | 0.004 |
| GR | MDA | 0.242 | 0.006 |
| SOD | CP | 0.125 | 0.164 |
| CAT | CP | 0.247 | 0.005 |
| GSH | CP | 0.072 | 0.420 |
| GPx | CP | -0.078 | 0.383 |
| GR | CP | -0.038 | 0.670 |
| MDA | CP | 0.107 | 0.233 |
| SOD | PC1_SOD | 0.693 | <0.001 |
| CAT | PC1_SOD | 0.127 | 0.158 |
| GSH | PC1_SOD | 0.496 | <0.001 |
| GPx | PC1_SOD | 0.670 | <0.001 |
| GR | PC1_SOD | 0.619 | <0.001 |
| MDA | PC1_SOD | 0.685 | <0.001 |
| CP | PC1_SOD | 0.142 | 0.113 |
| SOD | PC2_CAT | 0.085 | 0.343 |
| CAT | PC2_CAT | 0.723 | <0.001 |
| GSH | PC2_CAT | -0.046 | 0.609 |
| GPx | PC2_CAT | -0.203 | 0.023 |
| GR | PC2_CAT | -0.176 | 0.049 |
| MDA | PC2_CAT | 0.003 | 0.974 |
| CP | PC2_CAT | 0.809 | <0.001 |
| PC1_SOD | PC2_CAT | <0.001 | 1.000 |

**Table S2.** Pearson correlations between blood morphology and biochemical indices, and each of index with Principal Component two axes.

| row | column | cor | p |
| --- | --- | --- | --- |
| Lymphocytes | Monocytes | 0.353 | <0.001 |
| Lymphocytes | Eosinophils | 0.172 | 0.055 |
| Monocytes | Eosinophils | 0.301 | 0.001 |
| Lymphocytes | Basophils | 0.103 | 0.253 |
| Monocytes | Basophils | 0.290 | 0.001 |
| Eosinophils | Basophils | 0.298 | 0.001 |
| Lymphocytes | RBC | -0.023 | 0.801 |
| Monocytes | RBC | -0.232 | 0.009 |
| Eosinophils | RBC | 0.008 | 0.930 |
| Basophils | RBC | -0.188 | 0.035 |
| Lymphocytes | WBC | 0.732 | <0.001 |
| Monocytes | WBC | 0.520 | <0.001 |
| Eosinophils | WBC | 0.551 | <0.001 |
| Basophils | WBC | 0.353 | <0.001 |
| RBC | WBC | 0.010 | 0.911 |
| Lymphocytes | Ht | 0.036 | 0.688 |
| Monocytes | Ht | -0.179 | 0.044 |
| Eosinophils | Ht | -0.002 | 0.983 |
| Basophils | Ht | -0.125 | 0.165 |
| RBC | Ht | 0.561 | <0.001 |
| WBC | Ht | -0.008 | 0.925 |
| Lymphocytes | Hb | -0.055 | 0.544 |
| Monocytes | Hb | -0.181 | 0.043 |
| Eosinophils | Hb | -0.065 | 0.466 |
| Basophils | Hb | -0.064 | 0.477 |
| RBC | Hb | 0.606 | <0.001 |
| WBC | Hb | -0.054 | 0.548 |
| Ht | Hb | 0.721 | <0.001 |
| Lymphocytes | MCV | 0.055 | 0.537 |
| Monocytes | MCV | 0.153 | 0.087 |
| Eosinophils | MCV | -0.010 | 0.913 |
| Basophils | MCV | 0.148 | 0.098 |
| RBC | MCV | -0.811 | <0.001 |
| WBC | MCV | -0.017 | 0.850 |
| Ht | MCV | 0.028 | 0.756 |
| Hb | MCV | -0.222 | 0.012 |
| Lymphocytes | MCH | -0.019 | 0.836 |
| Monocytes | MCH | 0.124 | 0.166 |
| Eosinophils | MCH | -0.064 | 0.475 |
| Basophils | MCH | 0.189 | 0.034 |
| RBC | MCH | -0.684 | <0.001 |
| WBC | MCH | -0.054 | 0.550 |
| Ht | MCH | -0.039 | 0.669 |
| Hb | MCH | 0.164 | 0.067 |
| MCV | MCH | 0.801 | <0.001 |
| Lymphocytes | MCHC | -0.115 | 0.199 |
| Monocytes | MCHC | -0.050 | 0.576 |
| Eosinophils | MCHC | -0.088 | 0.330 |
| Basophils | MCHC | 0.052 | 0.562 |
| RBC | MCHC | 0.230 | 0.010 |
| WBC | MCHC | -0.057 | 0.529 |
| Ht | MCHC | -0.104 | 0.245 |
| Hb | MCHC | 0.613 | <0.001 |
| MCV | MCHC | -0.351 | <0.001 |
| MCH | MCHC | 0.280 | 0.002 |
| Lymphocytes | Protein | 0.012 | 0.897 |
| Monocytes | Protein | 0.158 | 0.077 |
| Eosinophils | Protein | -0.035 | 0.696 |
| Basophils | Protein | 0.008 | 0.931 |
| RBC | Protein | -0.050 | 0.575 |
| WBC | Protein | -0.003 | 0.972 |
| Ht | Protein | -0.045 | 0.618 |
| Hb | Protein | 0.041 | 0.648 |
| MCV | Protein | 0.030 | 0.737 |
| MCH | Protein | 0.106 | 0.236 |
| MCHC | Protein | 0.116 | 0.195 |
| Lymphocytes | Urea | -0.029 | 0.750 |
| Monocytes | Urea | 0.074 | 0.408 |
| Eosinophils | Urea | 0.062 | 0.490 |
| Basophils | Urea | 0.126 | 0.161 |
| RBC | Urea | -0.005 | 0.957 |
| WBC | Urea | <0.001 | 0.998 |
| Ht | Urea | 0.050 | 0.577 |
| Hb | Urea | -0.087 | 0.333 |
| MCV | Urea | 0.047 | 0.603 |
| MCH | Urea | -0.069 | 0.441 |
| MCHC | Urea | -0.183 | 0.040 |
| Protein | Urea | 0.236 | 0.008 |
| Lymphocytes | Uric_acid | -0.080 | 0.375 |
| Monocytes | Uric_acid | 0.008 | 0.932 |
| Eosinophils | Uric_acid | 0.054 | 0.548 |
| Basophils | Uric_acid | 0.150 | 0.093 |
| RBC | Uric_acid | 0.098 | 0.273 |
| WBC | Uric_acid | -0.045 | 0.614 |
| Ht | Uric_acid | 0.111 | 0.214 |
| Hb | Uric_acid | 0.052 | 0.564 |
| MCV | Uric_acid | -0.039 | 0.665 |
| MCH | Uric_acid | -0.074 | 0.412 |
| MCHC | Uric_acid | -0.052 | 0.560 |
| Protein | Uric_acid | 0.148 | 0.097 |
| Urea | Uric_acid | 0.720 | <0.001 |
| Lymphocytes | TG | -0.126 | 0.159 |
| Monocytes | TG | -0.088 | 0.328 |
| Eosinophils | TG | 0.027 | 0.764 |
| Basophils | TG | 0.164 | 0.067 |
| RBC | TG | 0.046 | 0.606 |
| WBC | TG | -0.165 | 0.065 |
| Ht | TG | 0.089 | 0.321 |
| Hb | TG | 0.175 | 0.050 |
| MCV | TG | 0.011 | 0.905 |
| MCH | TG | 0.109 | 0.226 |
| MCHC | TG | 0.150 | 0.094 |
| Protein | TG | 0.525 | <0.001 |
| Urea | TG | 0.332 | <0.001 |
| Uric_acid | TG | 0.385 | <0.001 |
| Lymphocytes | Cholesterol_total | 0.024 | 0.791 |
| Monocytes | Cholesterol_total | 0.052 | 0.561 |
| Eosinophils | Cholesterol_total | -0.028 | 0.760 |
| Basophils | Cholesterol_total | -0.054 | 0.547 |
| RBC | Cholesterol_total | 0.053 | 0.556 |
| WBC | Cholesterol_total | 0.005 | 0.957 |
| Ht | Cholesterol_total | 0.017 | 0.849 |
| Hb | Cholesterol_total | 0.018 | 0.846 |
| MCV | Cholesterol_total | -0.053 | 0.559 |
| MCH | Cholesterol_total | -0.044 | 0.622 |
| MCHC | Cholesterol_total | 0.015 | 0.869 |
| Protein | Cholesterol_total | 0.751 | <0.001 |
| Urea | Cholesterol_total | 0.220 | 0.013 |
| Uric_acid | Cholesterol_total | 0.156 | 0.081 |
| TG | Cholesterol_total | 0.284 | 0.001 |
| Lymphocytes | HDL | 0.194 | 0.030 |
| Monocytes | HDL | 0.063 | 0.481 |
| Eosinophils | HDL | 0.018 | 0.843 |
| Basophils | HDL | -0.103 | 0.249 |
| RBC | HDL | 0.050 | 0.577 |
| WBC | HDL | 0.172 | 0.054 |
| Ht | HDL | 0.076 | 0.395 |
| Hb | HDL | 0.071 | 0.432 |
| MCV | HDL | -0.010 | 0.911 |
| MCH | HDL | 0.006 | 0.947 |
| MCHC | HDL | 0.026 | 0.773 |
| Protein | HDL | 0.571 | <0.001 |
| Urea | HDL | 0.094 | 0.296 |
| Uric_acid | HDL | 0.013 | 0.884 |
| TG | HDL | 0.066 | 0.465 |
| Cholesterol_total | HDL | 0.792 | <0.001 |
| Lymphocytes | LDL | -0.074 | 0.409 |
| Monocytes | LDL | 0.044 | 0.624 |
| Eosinophils | LDL | -0.138 | 0.123 |
| Basophils | LDL | -0.170 | 0.058 |
| RBC | LDL | -0.020 | 0.822 |
| WBC | LDL | -0.099 | 0.272 |
| Ht | LDL | -0.100 | 0.265 |
| Hb | LDL | -0.119 | 0.184 |
| MCV | LDL | -0.049 | 0.586 |
| MCH | LDL | -0.083 | 0.356 |
| MCHC | LDL | -0.051 | 0.572 |
| Protein | LDL | 0.429 | <0.001 |
| Urea | LDL | -0.167 | 0.061 |
| Uric_acid | LDL | -0.225 | 0.011 |
| TG | LDL | -0.141 | 0.116 |
| Cholesterol_total | LDL | 0.668 | <0.001 |
| HDL | LDL | 0.435 | <0.001 |
| Lymphocytes | Aspat | 0.068 | 0.448 |
| Monocytes | Aspat | 0.051 | 0.571 |
| Eosinophils | Aspat | -0.030 | 0.741 |
| Basophils | Aspat | -0.059 | 0.511 |
| RBC | Aspat | 0.108 | 0.231 |
| WBC | Aspat | 0.034 | 0.702 |
| Ht | Aspat | 0.075 | 0.406 |
| Hb | Aspat | 0.042 | 0.644 |
| MCV | Aspat | -0.077 | 0.394 |
| MCH | Aspat | -0.095 | 0.289 |
| MCHC | Aspat | -0.024 | 0.791 |
| Protein | Aspat | 0.619 | <0.001 |
| Urea | Aspat | 0.399 | <0.001 |
| Uric_acid | Aspat | 0.277 | 0.002 |
| TG | Aspat | 0.203 | 0.022 |
| Cholesterol_total | Aspat | 0.751 | <0.001 |
| HDL | Aspat | 0.534 | <0.001 |
| LDL | Aspat | 0.252 | 0.004 |
| Lymphocytes | Alat | 0.035 | 0.699 |
| Monocytes | Alat | 0.162 | 0.069 |
| Eosinophils | Alat | -0.075 | 0.403 |
| Basophils | Alat | -0.067 | 0.455 |
| RBC | Alat | -0.013 | 0.885 |
| WBC | Alat | -0.011 | 0.900 |
| Ht | Alat | 0.077 | 0.394 |
| Hb | Alat | 0.011 | 0.901 |
| MCV | Alat | 0.069 | 0.443 |
| MCH | Alat | 0.026 | 0.776 |
| MCHC | Alat | -0.068 | 0.447 |
| Protein | Alat | 0.523 | <0.001 |
| Urea | Alat | 0.316 | <0.001 |
| Uric_acid | Alat | 0.276 | 0.002 |
| TG | Alat | 0.147 | 0.100 |
| Cholesterol_total | Alat | 0.596 | <0.001 |
| HDL | Alat | 0.425 | <0.001 |
| LDL | Alat | 0.254 | 0.004 |
| Aspat | Alat | 0.718 | <0.001 |
| Lymphocytes | Albumins | 0.022 | 0.811 |
| Monocytes | Albumins | 0.127 | 0.156 |
| Eosinophils | Albumins | -0.077 | 0.393 |
| Basophils | Albumins | -0.062 | 0.491 |
| RBC | Albumins | -0.020 | 0.825 |
| WBC | Albumins | -0.050 | 0.577 |
| Ht | Albumins | -0.013 | 0.885 |
| Hb | Albumins | 0.023 | 0.796 |
| MCV | Albumins | 0.017 | 0.853 |
| MCH | Albumins | 0.052 | 0.560 |
| MCHC | Albumins | 0.054 | 0.546 |
| Protein | Albumins | 0.913 | <0.001 |
| Urea | Albumins | 0.254 | 0.004 |
| Uric_acid | Albumins | 0.161 | 0.071 |
| TG | Albumins | 0.373 | <0.001 |
| Cholesterol_total | Albumins | 0.825 | <0.001 |
| HDL | Albumins | 0.642 | <0.001 |
| LDL | Albumins | 0.493 | <0.001 |
| Aspat | Albumins | 0.726 | <0.001 |
| Alat | Albumins | 0.653 | <0.001 |
| Lymphocytes | PC1_biochemical | 0.023 | 0.797 |
| Monocytes | PC1_biochemical | 0.103 | 0.250 |
| Eosinophils | PC1_biochemical | -0.068 | 0.449 |
| Basophils | PC1_biochemical | -0.066 | 0.463 |
| RBC | PC1_biochemical | 0.061 | 0.495 |
| WBC | PC1_biochemical | -0.019 | 0.833 |
| Ht | PC1_biochemical | 0.045 | 0.615 |
| Hb | PC1_biochemical | 0.051 | 0.574 |
| MCV | PC1_biochemical | -0.042 | 0.641 |
| MCH | PC1_biochemical | -0.025 | 0.780 |
| MCHC | PC1_biochemical | 0.028 | 0.754 |
| Protein | PC1_biochemical | 0.874 | <0.001 |
| Urea | PC1_biochemical | 0.368 | <0.001 |
| Uric_acid | PC1_biochemical | 0.280 | 0.001 |
| TG | PC1_biochemical | 0.388 | <0.001 |
| Cholesterol_total | PC1_biochemical | 0.934 | <0.001 |
| HDL | PC1_biochemical | 0.742 | <0.001 |
| LDL | PC1_biochemical | 0.543 | <0.001 |
| Aspat | PC1_biochemical | 0.813 | <0.001 |
| Alat | PC1_biochemical | 0.731 | <0.001 |
| Albumins | PC1_biochemical | 0.933 | <0.001 |
| Lymphocytes | PC2_morphology | -0.120 | 0.180 |
| Monocytes | PC2_morphology | -0.310 | <0.001 |
| Eosinophils | PC2_morphology | -0.053 | 0.556 |
| Basophils | PC2_morphology | -0.284 | 0.001 |
| RBC | PC2_morphology | 0.910 | <0.001 |
| WBC | PC2_morphology | -0.094 | 0.297 |
| Ht | PC2_morphology | 0.207 | 0.020 |
| Hb | PC2_morphology | 0.331 | <0.001 |
| MCV | PC2_morphology | -0.955 | <0.001 |
| MCH | PC2_morphology | -0.828 | <0.001 |
| MCHC | PC2_morphology | 0.237 | 0.008 |
| Protein | PC2_morphology | -0.107 | 0.233 |
| Urea | PC2_morphology | -0.058 | 0.516 |
| Uric_acid | PC2_morphology | 0.037 | 0.682 |
| TG | PC2_morphology | -0.031 | 0.732 |
| Cholesterol_total | PC2_morphology | 0.020 | 0.822 |
| HDL | PC2_morphology | -0.012 | 0.894 |
| LDL | PC2_morphology | 0.040 | 0.655 |
| Aspat | PC2_morphology | 0.054 | 0.552 |
| Alat | PC2_morphology | -0.093 | 0.298 |
| Albumins | PC2_morphology | -0.074 | 0.411 |
| PC1_biochemical | PC2_morphology | <0.001 | 1.000 |
| Lymphocytes | PC3_leukocytes | 0.698 | <0.001 |
| Monocytes | PC3_leukocytes | 0.602 | <0.001 |
| Eosinophils | PC3_leukocytes | 0.623 | <0.001 |
| Basophils | PC3_leukocytes | 0.456 | <0.001 |
| RBC | PC3_leukocytes | 0.096 | 0.283 |
| WBC | PC3_leukocytes | 0.910 | <0.001 |
| Ht | PC3_leukocytes | -0.026 | 0.771 |
| Hb | PC3_leukocytes | -0.112 | 0.211 |
| MCV | PC3_leukocytes | -0.131 | 0.144 |
| MCH | PC3_leukocytes | -0.214 | 0.016 |
| MCHC | PC3_leukocytes | -0.125 | 0.164 |
| Protein | PC3_leukocytes | -0.011 | 0.905 |
| Urea | PC3_leukocytes | 0.156 | 0.082 |
| Uric_acid | PC3_leukocytes | 0.127 | 0.158 |
| TG | PC3_leukocytes | -0.101 | 0.262 |
| Cholesterol_total | PC3_leukocytes | -0.014 | 0.878 |
| HDL | PC3_leukocytes | 0.125 | 0.165 |
| LDL | PC3_leukocytes | -0.199 | 0.025 |
| Aspat | PC3_leukocytes | 0.088 | 0.326 |
| Alat | PC3_leukocytes | 0.032 | 0.718 |
| Albumins | PC3_leukocytes | -0.038 | 0.675 |
| PC1_biochemical | PC3_leukocytes | <0.001 | 1.000 |
| PC2_morphology | PC3_leukocytes | <0.001 | 1.000 |
| Lymphocytes | PC4_nitogen | -0.209 | 0.019 |
| Monocytes | PC4_nitogen | -0.129 | 0.149 |
| Eosinophils | PC4_nitogen | 0.050 | 0.582 |
| Basophils | PC4_nitogen | 0.210 | 0.018 |
| RBC | PC4_nitogen | 0.110 | 0.220 |
| WBC | PC4_nitogen | -0.181 | 0.043 |
| Ht | PC4_nitogen | 0.247 | 0.005 |
| Hb | PC4_nitogen | 0.163 | 0.069 |
| MCV | PC4_nitogen | 0.046 | 0.608 |
| MCH | PC4_nitogen | 0.014 | 0.878 |
| MCHC | PC4_nitogen | -0.053 | 0.553 |
| Protein | PC4_nitogen | -0.014 | 0.877 |
| Urea | PC4_nitogen | 0.727 | <0.001 |
| Uric_acid | PC4_nitogen | 0.794 | <0.001 |
| TG | PC4_nitogen | 0.543 | <0.001 |
| Cholesterol_total | PC4_nitogen | -0.176 | 0.048 |
| HDL | PC4_nitogen | -0.306 | <0.001 |
| LDL | PC4_nitogen | -0.604 | <0.001 |
| Aspat | PC4_nitogen | 0.126 | 0.160 |
| Alat | PC4_nitogen | 0.114 | 0.203 |
| Albumins | PC4_nitogen | -0.057 | 0.525 |
| PC1_biochemical | PC4_nitogen | <0.001 | 1.000 |
| PC2_morphology | PC4_nitogen | <0.001 | 1.000 |
| PC3_leukocytes | PC4_nitogen | <0.001 | 1.000 |
|  |  |  |  |
