# Supplementary material for: Relationships between pesticides, polychlorinated biphenyls, blood parameters and oxidative stress of white stork Ciconia ciconia chicks in Poland
Source: Environ Sci Pollut Res Int. 2024 Jun 26;31(31):43996–4004. doi: 10.1007/s11356-024-34072-5 (PMC11252220; doi:10.1007/s11356-024-34072-5)
Supplement: Supplementary file 3 — Supplementary file3 (DOCX 116 KB) [file 11356_2024_34072_MOESM3_ESM.docx]

**Supplementary materials**

**Figures**

**Fig. S1.** Spatial autocorrelation for beta HCH with Moran’s local indicator (I). Minimal value of this indicator is -1 and means perfect dispersion, 0 means random dispersion and high cluttering would be close to 1.

**Fig. S2.** Spatial autocorrelation for DDE with Moran’s local indicator (I). Minimal value of this indicator is -1 and means perfect dispersion, 0 means random dispersion and high cluttering would be close to 1.

**Fig. S3.** Spatial autocorrelation for PCB with Moran’s local indicator (I). Minimal value of this indicator is -1 and means perfect dispersion, 0 means random dispersion and high cluttering would be close to 1.

**Fig. S4.** Correlation plot between enzymes, and each of enzyme with Principal Component two axes. PC1 axis is positively correlated with SOD, MDA and GSH (hereafter PC1_SOD). PC2 axis is positively correlated with CAT and CP enzyme (hereafter called PC2_CAT). Right scale indicates Pearson correlation.

**Fig. S5.** Correlation plot between blood morphology and biochemical indices, and each of index with Principal Component two axes. Right scale indicates Pearson correlation.
